# Progressing the field of Regenerative Rehabilitation through novel interdisciplinary interaction

**DOI:** 10.1038/s41536-020-00102-2

**Published:** 2020-09-23

**Authors:** Victor Cheuy, Silvia Picciolini, Marzia Bedoni

**Affiliations:** 1 Department of Physical Therapy and Rehabilitation Science, University of California San Francisco, San Francisco, CA USA; 2 Department of Radiology and Biomedical Imaging, University of California San Francisco, San Francisco, CA USA; 3 IRCCS Fondazione Don Carlo Gnocchi, Milan, Italy

**Keywords:** Tissue engineering, Regeneration, Translational research

## Abstract

The synergy between biological and bioengineering advances is critical to developing novel and impactful translational therapies. However, there currently are few opportunities for regenerative scientists to be exposed to the methodologies commonly employed in the clinic by rehabilitation professionals, and most rehabilitation scientists and clinicians are not exposed to the many advances of regenerative medicine. This disconnect has impeded the pace of progress in the field. The Eighth Annual International Symposium on Regenerative Rehabilitation brought together basic scientists, engineers, and rehabilitation clinicians to present scientifically rigorous and cutting-edge research and clinical management, focusing on new and innovative approaches that combine discoveries in tissue engineering, medical devices, and cellular therapies with rehabilitative protocols.

## Introduction

Regenerative Rehabilitation seeks to optimize patient outcomes through an integration of two fields: regenerative medicine and rehabilitation science. The former focuses on tissue repair or replacement due to loss from injury, disease, or age. This is achieved primarily through the enhancement of endogenous stem cell function or the transplantation of exogenous stem cells. The latter focuses on the use of mechanical and other stimuli to promote functional recovery. This synergy between biological and bioengineering advances is critical to developing novel and impactful translational therapies^1^. However, there currently are few opportunities for regenerative scientists to be exposed to the methodologies commonly employed in the clinic by rehabilitation professionals. Conversely, most rehabilitation scientists and clinicians are not exposed to the many advances of regenerative medicine. This disconnect has impeded the pace of progress in the field. To this end, the International Consortium for Regenerative Rehabilitation—comprised of 16 institutions—aims to increase interdisciplinary interaction. Thus, as technologies are developed and our understanding of regenerative biology progresses, advances may be efficiently translated to the clinic^2^.

To address this need, the annual international symposium is designed to propel the translation of regenerative technologies into functionally relevant treatment interventions that have the potential to transform rehabilitative healthcare. The Eighth Annual International Symposium on Regenerative Rehabilitation brought together basic scientists, engineers, and rehabilitation clinicians in order to create a fertile ground for discussion, interaction, and networking across disciplines. The symposium took place on 24–26 October 2019, in Charlottesville, Virginia, and was co-hosted by the University of Virginia and the Uniformed Services University of the Health Sciences.

The symposium held sessions to address three principle objectives: (1) *Scientific*: to promote the clinical translation of scientific discoveries in the field of regenerative medicine by communicating and disseminating research findings that demonstrate the synergistic relationship between regenerative medicine and rehabilitation; (2) *Programmatic*: to provide multiple forums in which scientists, rehabilitation clinicians, and trainees may interact, exchange ideas, and identify novel research directions relating to the field of Regenerative Rehabilitation; (3) *Mentorship and training*: to introduce the concept of Regenerative Rehabilitation to promising graduate students, medical residents, clinical fellows, post-doctoral research fellows, and other junior investigators and clinicians, inspiring and supporting them to embrace and incorporate innovative technologies in their nascent clinical practices and research programs.

## Scientific sessions

The scientific sessions brought together world-renowned researchers and clinicians to present on scientifically rigorous and cutting-edge research and clinical management. Topics focused on new and innovative approaches that combine discoveries in tissue engineering, medical devices, and cellular therapies with rehabilitative protocols.

Drs. A. Bobby Chhabra and Fred Epstein from the University of Virginia opened the meeting by emphasizing the importance of interdisciplinary collaborations in research. Opportunities like this symposium do not just highlight existing teamwork but foster new engagement and relationships that are critical to translational success. Picking up on this theme, Dr. Anthony Atala (Wake Forest Institute for Regenerative Medicine) shared his journey through a three-decade long research career in tissue and organ engineering, including the successful implantations of tissue engineered kidneys, bladders, and sexual organs^3–5^. His work has pioneered the use of material-alone, cell-alone, and material+cell approaches to address tissue defects of varying size as well as across all four levels of tissue complexity (e.g., flat, tubular, hollow non-tubular, solid organs)^6^. Dr. Atala highlighted two important lessons learned through his research. First, is the value of a methodical approach. The genesis of each line of investigation is thorough cell biology and materials sciences studies, then transitioning to small, medium, and large animal studies. Only after which does it reach human patients for testing and long-term follow-up. Second, is the necessity of a multidisciplinary team—including cell and molecular biologists, material scientists, biochemists, and physicians—where team science has helped propel this technology from the bench to the bedside. Dr. Atala also discussed work in the cell therapy area, such as the use of skeletal muscle for shoulder cuff injuries, and concluded by sharing his future directions in stem cell therapy, specifically the use of amniotic fluid and placental stem cells^7^. He emphasized their clinical utility with respect to ease of collection, shared properties with embryonic and adult stem cells, lack of tumor potential and rejection concerns, and storage capabilities.

Plenary speaker Dr. Robert Guldberg (University of Oregon) provided an insightful talk on the preclinical and in vivo models his lab uses to assess vascular and bone growth under different load conditions, with both acute and delayed treatment models. His group has pioneered the integration of implantable wireless sensors to monitor the mechanical environment within the regenerative niche^8^. Indeed, the new vascular network formation is very sensitive to biomechanical conditions, but the regulatory role of matrix deformations and remodeling during tissue regeneration in vivo needs to be evaluated. He showed how functional loading has a potent time-dependent effect on both mineralization and vascular growth in vivo, with early loading being detrimental whereas delayed loading enhanced outcomes^9^. Additionally, in a controlled, in vitro model, Dr. Guldberg and his group have demonstrated that allowing the vascular network to develop for 5 days before adding compression plus shear optimized outcomes. Dr. Guldberg’s Regenerative Rehabilitation approach to large bone defect repair nicely demonstrated the clinical relevance of timing and method of stimulation in designing rehabilitation protocols.

### Cellular rehabilitation: maximizing stem cell function

This session focused on the role of mechanical stimulation for enhancing stem cell function. In 2006, Dr. Adam Engler (University of California, San Diego) and colleagues first demonstrated that stem cells are highly mechanosensitive and that biophysical characteristics of the extrinsic microenvironment is a potent driver of stem cell lineage specification^10^. As an extension of this pioneering work, Dr. Engler kicked off the session with an engaging presentation on leveraging biomaterials to better approximate extracellular matrix properties to induce cardiomyocyte phenotypes. This disease-in-a-dish approach allows for induction of pathological cardiomyocytes responses to study, identify, and subsequently interrogate signaling pathways, which may ultimately inform clinical interventions. These interesting results suggest that any culture system should require specific dynamic materials that change as the niche does in vivo. Dr. Fabrisia Ambrosio (University of Pittsburgh) then shared her work on extracellular vesicles and the protein Klotho. Understanding how circulating factors affect the mitochondrial function of muscle stem cells provides insight into how muscle loses its ability to heal with increasing age, which could present an interventional target to restore one’s molecular profile to a more “youthful” stage.

Dr. Robert Grange (Virginia Tech) talked about the pathogenic mechanisms and treatments for Duchenne muscular dystrophy (DMD), with a focus on rescuing dystrophic muscle so patients can better perform and enjoy activities of daily living. His Regenerative Rehabilitation approach was to use gene therapy in combination with endurance exercise in a rodent model—the mdx mouse—to significantly improve the time-to-fatigue during the final treadmill run at the end of the training period. Dr. Grange has previously shown that mdx muscles can functionally adapt to chronic exercise, but exercise alone is not enough to completely overcome the maladaptive response of dystrophic muscle^11^. The results demonstrated that, although gene therapy alone can improve endurance, muscles in the mdx mouse model treated with gene therapy and combined with exercise are capable of adapting to training and achieving significant gains. Consequently, this Regenerative Rehabilitation therapeutic approach has great clinical potential for development of effective drug and physical therapy treatments of DMD.

### Celebrating 5 years of AR^3^T: launching new lines in Regenerative Rehabilitation research

Co-director Dr. Thomas Rando (Stanford University) shared an overview of the Alliance for Regenerative Rehabilitation Research & Training (AR^3^T), an NIH-funded resource network established in 2015. The mission of AR^3^T is to support the expansion of scientific knowledge, expertise, and methodologies across the fields of regenerative medicine, biophysics and rehabilitation. To fully realize this goal, AR^3^T promotes education, training, research support, and funding opportunities (www.ar3t.pitt.edu). Dr. Rando reiterated AR^3^T’s support of the Symposium and described the broad range of opportunities that are available through AR^3^T to help researchers develop and succeed with innovative lines of Regenerative Rehabilitation research. The opportunities include short-term sabbaticals to AR3T sites, consultations with subject matter experts, pilot funding, technology development grants, paired mentoring experiences, and access to diverse educational courses.

To further highlight the effectiveness of this P2C grant-funded center, past grant awardees Drs. Spencer Szczesny (Pennsylvania State University), Dr. Sunil Gandhi (University of California, Irvine), and Dr. Gunes Uzer (Boise State University) were selected to present on their AR^3^T-funded research findings. In line with the Symposium’s objective of promoting Regenerative Rehabilitation by junior investigators, Dr. Szczesny shared his research on the interplay between tendon multiscale mechanics and mechanobiology in the context of tissue remodeling. Dr. Szczesny’s multidisciplinary research involves the use of multiscale mechanical testing, cell and tissue culture, and biomaterial fabrication. The goal of this work is to build a computational model of growth and remodeling that will allow for prediction of loading protocols that lead to degeneration versus regenerative repair.

Dr. Gandhi discussed his progress in the development of a method for live imaging of somatosensory fibers in cervical spinal cord that can help to determine whether effective human neural stem cell engraftment enhances robotic-assisted therapy after spinal cord injury, thus improving recovery. Dr. Uzer showed a 3D printable, repeatable, adaptable and easy-to-use model of bone marrow that will be useful for studying cell responses in specific mechanical environments.

### Atten-hut!: Impact of Regenerative Rehabilitation on warfighter care

The Department of Defense (DoD) has increasingly relied on the role of Regenerative Rehabilitation to advance the translation of cutting-edge regenerative technologies for the functional recovery of severely wounded soldiers and civilians. This is evidenced by recent funding opportunities such as the RESTORE effort that calls for Regenerative Rehabilitation research and the AFIRM effort to advance regenerative medicine. Along these lines, Colonel Sarah Goldman (U.S. Army Medical Research and Development Command) illustrated how war has motivated medical advances and she discussed applicable DoD medical research funding opportunities, including opportunities within the Congressionally Directed Medical Research Programs and other DoD Medical Research efforts.

The plenary speaker of this session was Dr. Robert Mauck (University of Pennsylvania), sharing his translational mechanobiology work. He focused on how to motivate cell migration towards a wound site and how to use mechano-activation to our advantage for improved factor delivery to promote formation and repair of musculoskeletal tissue^12^.

Dr. John Houle (Drexel University College of Medicine) shared his research on the duality of exercise after spinal cord injury (SCI), which can promote neural plasticity but also block aberrant neuroplasticity^13^. More specifically, demonstrating the importance of a Regenerative Rehabilitation approach, he showed that regeneration of neurons in the cord following SCI was significantly increased with a combination of exercise and cell graft.

Dr. David Weiss (University of Virginia) brought a valuable orthopedic surgeon’s perspective to the session. He discussed the challenges in trauma surgery when dealing with volumetric muscle loss (VML), how to evaluate the injury, and the long path ahead to regenerating muscle for massive loss injuries. He described UVA’s collaborative approach for studying VML, which includes working with the Biomedical Engineering Department to quantify the location and magnitude of VML and with the Kinesiology Department to perform functional testing.

### In the clinic: stem cell therapies for MSK rehabilitation

This session began with plenary speaker Dr. George Christ (University of Virginia), who focused on volumetric muscle loss and the profound functional deficit that can be disproportionate to the volume lost^14^. He also shared the multiscale biomechanical approaches currently being used to understand and ideally develop new rehabilitative approaches and therapeutics to reverse it (i.e., computational modeling, muscle function testing, imaging, kinematics, and kinetics). Drs. Joanne Borg-Stein (Harvard Medical School) and Kenneth Mautner (Emory University) both shared their esteemed insights into bone marrow aspirate and adipose-derived stem cells. There are clinical logistics to consider with respect to the patient and provider, and certain rehabilitation protocols can be used to optimize the precision of regenerative medicine. Gaps in clinical practice using regenerative medicine approaches include identifying the best cell source, correct dose, best route of delivery, and optimal timing and type of rehabilitation. This led to an excellent panel discussion led by Dr. Borg-Stein, Dr. Mautner, Dr. Nelson Hager (Uniformed Services University of the Health Sciences), and Dr. Quanjun Cui (University of Virginia). Topics included the clinical rationale behind using bone marrow versus adipose, the role of age in deciding treatment approach, what factors may play a role in responding to treatment, and the need to establish standards for stem cell therapeutics. This session’s clinical focus brought home the importance of collaborating in the translational process so that research data can be used to inform rehabilitation protocols to be used in combination with regenerative medicine therapies.

### New horizons: Regenerative Rehabilitation takes an eye to the clinic

This session provided examples of translational research in the field. Dr. Michael Boninger (University of Pittsburgh) discussed the application of assistive technologies to increase function and participation in individuals with neurological disabilities. This is a very crucial topic that is reported to be of relevant impact in different aspects of neurological patient lives, such as in health, psychological and social status, education, and motor or cognitive learning^15^. Importantly, he challenged attendees to consider how neural technologies could be combined with regenerative medicine approaches in the brain and spinal cord to enhance stem cell differentiation and the formation of new connections that will result in improved healing.

Dr. Kacey Marra (University of Pittsburgh) shared her authentic, humorous, and valuable perspective on what the translational research timeline looked like for her AxoMax technology, detailing the 20-year buildup from idea to human clinical trials to forming a company^16^. The idea of the AxoMax technology is to use a biodegradable polymer tube containing a drug delivery system to promote the growth of peripheral nerves over large gaps (>3 cm), overcoming the current clinical limitations in nerve repair. One future direction is to add electrical stimulation and exercise to maximize outcomes.

Dr. Silvia Blemker (University of Virginia) shared her collaborative research on 3D muscle modeling, which provides insight into the form, function, and biology of muscle. This work has wide-reaching applications for studying atrophy, injury, and dystrophy, as well as to inform the design and testing of tissue engineering technologies for muscle repair.

Dr. Shawn Russell (University of Virginia) spoke on the functional assessment of biomechanics, where the kinematics and kinetics of animal locomotion can be powerful tools for quantifying functional change after volumetric muscle loss^17^. Understanding these altered movement strategies will better inform the design of regenerative and rehabilitation approaches to prevent the development of chronic movement compensations.

## Programmatic sessions

Over the past 8 years, the symposium series has evolved to meet the needs of such a disparate audience. While a strength of the symposium is in bringing so many different students, researchers, and clinicians together in one place, there was also a need to program in targeted learning opportunities to meet specific needs.

A Clinical Special Interest Group (SIG), formed in response to the growing number of rehabilitation clinicians, provides an opportunity to meet and share experiences related to the treatment of regenerative medicine therapies in the clinic. The Clinical SIG breakfast discussion, led by Drs. Carmen Terzic (Mayo Clinic Rochester) and Quanjun Cui, allowed attendees to share ideas and strategies for clinical approaches following regenerative medicine therapies to optimize outcomes.

Held for the first time at the symposium, a breakfast discussion led by the University of Pittsburgh’s Dr. Fabrisia Ambrosio and Mrs. Paula Davis centered on diversity and inclusion. Attendees shared multiple perspectives and thoughts on supporting diversity in the student body and at the faculty level. Recommendations that resulted from the breakfast included that we should all obtain training on diversity and implicit bias, as each one of us can be an ally, and to support diversity across the STEM pipeline.

Post-symposium workshops were “Clinical Study Design for Regenerative Rehabilitation” led by Dr. Marcas Bamman (University of Alabama), and “Rehabilitation Strategies in Preclinical Models” led by Dr. Linda Noble-Haeusslein (University of Texas, Austin). The goal of Dr. Bamman’s workshop was to address challenges and current opportunities specific to clinical trials in medical rehabilitation. These included trial design, issues in recruitment and retention, funding opportunities, and mobile technology integration into rehabilitation trials. An introduction to clinical trial resources for investigators was given, specifically the REACT Center and the Medical Rehabilitation Research Resource (MR3) Network Coordinating Center. The goals of Dr. Noble-Haeusslein’s workshop were to overview preclinical research design using a rodent model and to cover methods of rehabilitation and outcome measurements. Together, these will help optimize basic regenerative rehabilitation research for more successful translations of basic research to the clinic.

## Mentorship and training sessions

A pre-symposium workshop was led by Dr. Rosemarie Hunziker—a former program director of the National Institute of Biomedical Imaging and Bioengineering—on how to maximize success in NIH grant writing. Geared towards trainees, early investigators, and those who may not be familiar with NIH funding, Dr. Hunziker provided an in-depth and entertaining crash course on the tips, tricks, and common challenges of the NIH.

A poster session provided an excellent networking opportunity between senior researchers, clinician scientists, and students. A vast spectrum of topics was presented, including implantable sensors, biomaterials, molecular and cellular responses to biophysical signals, and mechanical stimulation in cellular therapeutics and tissue engineering.

## Conclusion

This symposium featured world-renowned researchers and clinicians, focusing on the emerging field of Regenerative Rehabilitation; it succeeded in creating a platform for bridging several areas of expertise in a setting that fosters discussion, interaction, cross-discipline pollination and networking. Drs. Paul George (Stanford University) and Shailly Jariwala (Uniformed Services University of the Health Sciences) shared a success story of how networking at last year’s Symposium led to their collaborative project, submitted to the Department of Defense, using nerve conduits combined with electrical and chemical stimulation for the treatment of peripheral nerve injury. This session invited attendees to think of innovative approaches and collaborations to continue moving the field of Regenerative Rehabilitation forward. Looking ahead, the P2C grant supporting AR^3^T was selected for renewal and received its notice of award July 2020. Due to COVID-19, the AR^3^T Executive Leadership recently made the difficult decision to postpone the 2020 symposium to 4–6 November 2021 at the University of Texas, Austin. However, a number of virtual options are being planned in the interim that will tap into the pioneering aptitude of our community to deliver world-class education and spotlight Regenerative Rehabilitation innovation.

## Data Availability

Data sharing not applicable to this article type as no datasets were generated or analyzed.
